# Genotypic and phenotypic variability of 22q11.2 microdeletions – an institutional experience

**DOI:** 10.3934/molsci.2021020

**Published:** 2021-12-09

**Authors:** Gabrielle C. Manno, Gabrielle S. Segal, Alexander Yu, Fangling Xu, Joseph W. Ray, Erin Cooney, Allison D. Britt, Sunil K. Jain, Randall M. Goldblum, Sally S. Robinson, Jianli Dong

**Affiliations:** 1 School of Medicine, University of Texas Medical Branch, Galveston, Texas, USA; 2 Department of Pathology, University of Texas Medical Branch, Galveston, Texas, USA; 3 Department of Pediatrics, University of Texas Medical Branch, Galveston, Texas, USA

**Keywords:** genotype-phenotype correlation, chromosome 22q11.2, microdeletions, 22q11.2 deletion syndromes, chromosome microarray

## Abstract

Patients with chromosome 22q11.2 deletion syndromes classically present with variable cardiac defects, parathyroid and thyroid gland hypoplasia, immunodeficiency and velopharyngeal insufficiency, developmental delay, intellectual disability, cognitive impairment, and psychiatric disorders. New technologies including chromosome microarray have identified smaller deletions in the 22q11.2 region. An increasing number of studies have reported patients presenting with various features harboring smaller 22q11.2 deletions, suggesting a need to better elucidate 22q11.2 deletions and their phenotypic contributions so that clinicians may better guide prognosis for families. We identified 16 pediatric patients at our institution harboring various 22q11.2 deletions detected by chromosomal microarray and report their clinical presentations. Findings include various neurodevelopmental delays with the most common one being attention deficit hyperactivity disorder (ADHD), one reported case of infant lethality, four cases of preterm birth, one case with dual diagnoses of 22q11.2 microdeletion and Down syndrome. We examined potential genotypic contributions of the deleted regions.

## 1. Introduction

Chromosome 22q11.2 deletion syndromes result from deletion of various intervals at 22q11.2 region mediated by meiotic non-allelic homologous recombination of low copy repeats (LCRs) throughout this region termed LCRs A-H [1]. According to LCR deletion intervals, 22q11.2 deletion syndromes have been classified to different entities including proximal deletions (A-B, A-D, A-E, A-F), central deletions (B-D, C-D), and distal deletions (type I: C-E, D-E, D-F; type II: E-F; type III: D-H, E-H, F-H) [1]. For examples, DiGeorge syndrome (DGS, OMIM 188400) and velocardiofacial syndrome (VCFS, OMIM 192430) are caused by deletions spanning LCRs A-D interval, whereas chromosome 22q11.2 deletion syndrome, distal (OMIM 611867) harbors deletions spanning LCRs D-H [1]. Phenotype of 22q11.2 deletion syndromes is heterogenous and includes, but is not limited to variable cardiac defects, parathyroid and thyroid gland hypoplasia, immunodeficiency, velopharyngeal insufficiency, developmental delay, intellectual disability, cognitive impairment, and psychiatric disorders [1–6]. 22q11.2 deletion syndrome was traditionally detected as megabase (mb) deletions including *TBX1* gene in the region resulting in syndromic features of DGS and VCFS. However, an increasing number of studies using chromosome microarray (CMA) have reported a significant number of symptomatic patients harboring smaller 22q11.2 microdeletions, with some as small as 100 kilobase (kb), with or without *TBX1* gene [1,3,4,7]. Moreover, several studies involving DGS/VCFS patients diagnosed before genetic testing was widely available have reported an increasing variation of phenotypes, including non-classic phenotypes such as genitourinary abnormalities, prematurity, and skeletal defects [1,8–11].

To further delineate the genotype-phenotype associations, we performed an institutional retrospective case review of patients harboring 22q11.2 deletions detected by chromosomal microarray to determine concordance with reported genotype-phenotype correlation studies in addition to potentially identifying 22q11.2 subregions associated with novel phenotypes in pediatric patients.

## 2. Materials and methods

### 2.1. Subjects

From March 2013 to December 2019, approximately 2,000 germline CMA results were generated, and 16 patients were positive for 22q11.2 microdeletions. We performed chart reviews on these 16 patients using UTMB’s electronic medical record in the summer of 2020. Pediatric specialists examined the 16 patients, assigned phenotypes, made clinical diagnoses, and referred for CMA testing based on clinical features as detailed in Tables 1–3. This study was approved by the UTMB Institutional Review Board.

### 2.2. Chromosome microarray analysis

CMA was performed using peripheral blood and examined with Cytoscan HD microarray (Affymetrix, Santa Clara, CA). This array consists of 2,696,550 oligonucleotide probes, including 1,953,246 distinctive non-polymorphic oligonucleotide probes, and 743,304 single nucleotide polymorphism probes. Genomic DNA was extracted and purified from whole blood sample using Gentra Puregene Blood Kit (Qiagen Inc., Valencia, CA). Procedures for DNA digestion, adapter ligation, polymerase chain reaction (PCR), amplicon DNA fragmentation, labeling, and hybridization of the arrays were performed according to manufacturer’s instructions (Affymetrix, Santa Clara, CA). Results were investigated using the Chromosome Analysis Suite (ChAS; Affymetrix, Santa Clara, CA). The settings for smallest copy number variation (CNV) regions in ChAS were 25 kb and 25 probes for losses, and 50 kb and 50 probes for gains. Genomic linear positions are given relative to NCBI build 37 (hg19) [12]. Results were interpreted based on published literature, publicly available databases, and by investigating gene content following practice guidelines [13,14].

## 3. Results

**Figure 1.**
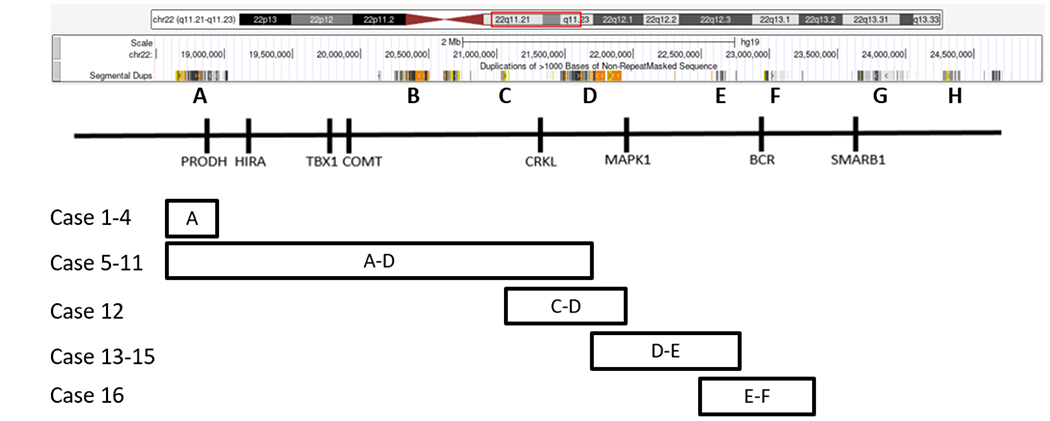
22q11.2 deletion regions in the sixteen cases. Key genes involved in the 22q11.2 deletion syndrome were according to Burnside, 2015 [1]. Diagram taken from UCSC genome browser (NCBI build 37 [hg19]). Letters A–H indicate low-copy repeats (LCRs) implicated in 22q11.2 deletion syndromes.

There were four A, seven A-D, one C-D, three D-E, and one E-F deletions (Figure 1, Table 1). Figure 1 maps these deletion regions with respect to previously investigated genes with major known contributions to 22q11.2 deletion syndrome phenotypes [1,3]. Tables 1–4 summarize the deletions and the main clinical manifestations of each patient. Cases 1–4 harbored heterozygous deletions approximately 108 kb, arr[GRCh37] 22q11.21(18,916,842–19,024,659)x1, flanked by LCR A (Figure 1, Table 1). Cases 1, 2, and 3 presented variable intellectual, behavioral, and psychomotor delays (Table 2). Two presented with attention deficit hyperactivity disorder (ADHD) and autism spectrum disorder (ASD). Cases 2–4 also presented with varying forms of craniofacial malformations, including submucous cleft palate, retrognathia, micrognathia, mandibular hypoplasia, and grade 2 microtia in the left ear resulting in hearing loss. Case 4 presented at birth with atrial secundum defect (ASD) which spontaneously resolved by age 3 (Tables 1–3).

Cases 5–11 harbored classic 22q11.2 deletions flanked by LCR A-D (Figure 1, Table 1). Case 5 had a one copy 2,821 kb deletion of 22: 18,644,790–21,465,659. The patient presented as a full-term male with a grade III/IV holosystolic murmur and found to have an ASD, ventricular septal defect (VSD), tricuspid insufficiency, and bilateral peripheral pulmonary stenosis. The patient also had frontal bossing and bilateral middle ear disorder. Low T cell receptor excision circles (TRECs) were present at birth. The patient presented multiple times with scabies, pinworms, otitis media, and oral candidiasis. By age 4, immunological studies demonstrated normal mitogen and pathogen response, normal CD4 and CD8 levels, eosinophilia, low IgM, and an absent IgG response to tetanus and candida. The patient also has significant psychomotor delay presenting as severe hypotonia, poor weight gain, and fine and gross motor delays (Tables 1–3).

Case 6 showed a 2,548 kb heterozygous deletion of 22: 18,916,842–21,465,662. The patient presented as a pre-term male infant born at 30 weeks with a submucous cleft palate and significant renal dysfunction. In utero ultrasound reported findings suggestive of polycystic kidney disease; at birth, his right kidney was aplastic with only 5% functional capacity and his left kidney was hypertrophic with 95% functionality. An underdeveloped scrotum and glandular hypospadias were also noted. The patient later had significant increase in appetite and subsequent weight gain, which is atypical as most individuals with 22q11 syndrome fall below the 15th percentile in weight [15]. This patient also presented with mixed receptive-expressive disorder, gross motor delay, intellectual disability, and ADHD. Throughout childhood, he presented multiple times with otitis media caused by Pseudomonas aeruginosa and Staphylococcus aureus, UTIs, and viral warts. He was also found to be positive for antinuclear antibodies (ANA). Atypical immunological testing included low CD4 and CD8 cells levels in addition to low IgM but high IgG and IgA levels. Genetic testing was ordered for suspicion of Prader-Willi syndrome but was negative.

Cases 7 and 8 shared a 2,884 kb single copy loss of 22: 18,916,842–21,800,797. Both presented with ASD, VSD, speech delays, growth failure, craniofacial malformations, especially hypertelorism. Specifically, case 8 presented with low lymphocytes, which self-resolved over an unspecified period. This patient had frequent infections with community respiratory viruses including respiratory syncytial virus. Case 8 specific defects included Tetralogy of Fallot (ToF) and a high arched palate. Case 8 presented with relatively more severe features including aplasia cutis, low set ears, growth failure, velopharyngeal incompetence, appendicular hypotonia, difficulty swallowing and controlling secretions, and left sided hearing loss (Tables 1–3).

Case 9 presented as a full-term male born with respiratory distress. Laryngoscopy demonstrated laryngomalacia, anterior pharyngeal webbing, subglottic stenosis, and congenital paralysis of the true vocal cords. Hypoparathyroidism, hypocalcemic seizures, and low TRECs were also present at birth. Polydactyly was also noted at birth. Due to suspected congenital abnormalities, CMA was utilized shortly after birth and reported a 2,999 kb deletion at 22: 18,916,842–21,915,509, which prompted further clinical evaluation. Subsequent findings included a small ASD and potential tricuspid regurgitation, and left pelviectasis. Pertinent immunological studies reported low CD3 and CD4 counts; B cell testing demonstrated response to candida antigen but not tetanus antigen. Mixed receptive expressive speech disorder was diagnosed. Moreover, throughout physician visits, the child had failure to thrive likely due to feeding difficulties from congenital abnormalities.

Case 10 presented as a 35-week 4 day old preterm who died 8 days after birth secondary to multiple intraventricular hemorrhages. The patient had several congenital abnormalities including a moderate-sized ASD, patent ductus arteriosus, and moderate right-sided atrial and ventricular dilatation, multicystic dysplastic kidney, adrenal hyperplasia, and hypocalcemia. CMA revealed 2,884 kb loss at 22: 18,916,842–21,800,797.

Case 11 presented as a 32-week-old preterm infant girl born with respiratory distress. CMA revealed a 3,152 kb loss at 22: 18,648,866–21,800,797 in the infant girl. The pregnancy was complicated with anhydramnios. The infant presented with moderate to large ASD and right-sided heart enlargement, aortic thickening, microcephaly, low birth weight (1,600 g, 3 lb 4 oz), and hypocalcemic seizures. The infant had failure to thrive suspected secondary to poor feeding. The child also presented with low IgM levels. The family history is remarkable for mother and a maternal half-brother with 22q11.2 deletion syndrome; however, the size and deleted region were not provided. Mother reported a personal history of kidney stones and the half-brother with a single kidney.

Case 12 was a full-term male whose pregnancy was complicated by maternal cannabis and tobacco use and a urinary tract infection (UTI) at time of delivery. Following a cesarean section, the infant presented with a weak cry which prompted laryngoscopy that demonstrated anterior glottic webbing and subglottic stenosis. Further evaluation revealed a bifid uvula and a notched hard palate. CMA revealed a 749 kb LCR C-D deletion, 22: 21,049,799–21,798,907. Cardiac evaluation revealed a dilated aortic root, coronary sinus, and trace tricuspid insufficiency. Poor weight gain was noted at his 15-month visit, falling from the 50th percentile to <10th percentile. This trend continued throughout childhood, never exceeding the 15th percentile with no clear attributable causes. Endocrine testing revealed elevated TSH and anti-thyroid peroxidase antibodies, resulting in a diagnosis of Hashimoto’s disease. Further, hydronephrosis secondary to bilateral pelviectasis was diagnosed at age 3 years.

Cases 13–15 presented with deletions encompassing LCR D-E, 22: 21,465,661–22,962,196. Case 13 presented with structural cardiac abnormalities including a small ASD and tricuspid insufficiency. This patient was later diagnosed with growth delay, speech delay, intellectual disability with regression, autism spectrum disorder, and ADHD. Cases 14 and 15 were familial deletions. The mother, case 15, presented with mild seborrheic dermatitis, ADHD, and self-reported low lymphocytes and recurrent upper respiratory infections (URIs) and otitis media in childhood. Her son, case 14, presented with a more severe phenotype including global developmental delay, intellectual disability, and ADHD. In addition, he had left sided hearing loss with external canal atresia, a preauricular tag, severe muscular hypotonia, brachycephaly, hypotelorism, bilateral single transverse palmar crease, a broad nasal bridge, a low white blood cell count, and recurrent URIs.

Case 16 presented as a female infant harboring a 687 kb LCR E-F deletion, 22: 22,962,196–23,649,155 in addition to trisomy 21 (Down syndrome). The pregnancy was complicated by polyhydramnios. At birth, she had persistent pulmonary hypertension of the newborn secondary to complete atrioventricular septal defect, large inlet VSD, large primum ASD, atrioventricular valve regurgitation, small right ventricular cavity size with right ventricular hypertrophy. She had congenital thrombocytosis which resolved within a month leading to a suspected transient abnormal myelopoiesis associated with Down syndrome. Though a developmental delay was noted, the patient was lost to follow up at 6 months of age and additional information is not available.

## 4. Discussion

We report a cohort of 16 cases harboring variable 22q11.2 deletions including 9 atypical deletions (4 A, 1 C-D, 3 D-E, 1 E-F) and 7 typical A-D deletions (Figure 1). Approximately 85–90% of individuals with 22q11.2 deletion syndromes have been reported to harbor LCR A-D deletions [1,3]. The reason for the high number of atypical deletions in relation to the number of typical deletions in our cases is unclear. One factor may relate to ascertainment bias. Patients with developmental disabilities, congenital anomalies, and general suspicion of chromosomal abnormalities may be referred to our CMA assay that can detect both typical and atypical deletions, whereas patients with more severe phenotype suspicious of DGS/VCFS might have been referred for karyotyping and FISH assays that more likely detect typical and large deletions [3,10,16,17].

This is a single institution study with limited number of cases. The 16 cases harbor variable 22q11.2 deletions presenting with variable clinical features, concordant with previous studies [1,18–21]. Interestingly, glottic web was found in cases 9 (A-D deletion) and 12 (C-D deletion), suggesting the contributing genetic factor(s) in the smaller LCR C-D region. Cases 9 and 11 both harbor proximal A-D deletions and presented with inguinal hernia requiring gastrostomy tubes. Cases 14 and 15 (LCR D-E) are son-mother couple. Consistent with previous report [22], Case 14 presented with more severe phenotypes than his mother (Case 15) who was identified as affected by 22q11.2 deletion syndrome after the birth of her son and presented ADHD that likely associated with the deletion. Although rare [19], this mother-son pair shows that distal deletions can be inherited. Case 11 (LCR A-D) is assumed maternal inheritance; however, the maternal test result is not provided. The origins of deletions in the other cases are unknown (Table 2). Case 16 is unusual who has Down syndrome in addition to distal E-F deletion presenting with a severe endocardial cushion defect found in more than half of Down syndrome patients [23]. This presentation suggests dominant cardiac effects of trisomy 21 over the 22q11.2 LCR E-F deletion [1,3,4]. Nonetheless, it is possible the LCR E-F deletion contributed to the severity of the endocardial cushion defects as this has been reported as a phenotype associated with deletions within this region [1]. Future studies may compare clinical features of trisomy 21 patients with and without 22q11.2 deletions.

Deletions flanking LCR A are found in cases 1–4 (Figure 1). The clinical significance of these deletions is uncertain. LCR A is also termed LCR22A-+A and found in both patients and controls and considered probably benign [24]. However, two patients are reported in the ClinGen database with heterozygous deletions in the small LCR A region as our cases 1–4, both interpreted as Pathogenic. Deletion in case nssv577839 (chr22: 18,890,271–18,999,862) is paternal origin and associated with abnormal facial shape, abnormality of limb bone morphology, cleft palate, flexion contracture, micrognathia, rocker bottom foot, scoliosis, webbed neck, and wide nasal bridge. Deletion in case nssv577840 (chr22: 18,905,109–19,015,451) is maternal origin and associated with protruding ear (clinicalgenome.org, last accessed October 26, 2021). Refseq genes in this region include *FAM230F*, *DGCR6*, *PRODH*, *DGCR5*, with pathogenic *PRODH* variants associated with autosomal recessive hyperprolinemia type I or autosomal susceptibility to schizophrenia [25–30]. A recent case control study reported microdeletions encompassing the *PRODH* and *DGCR6* genes to be a strong risk factor for hyperprolinemia type 1 but not for autism spectrum disorder suggesting more emphasis on the other lesser known *FAM230F* and *DGCR5* genes [31]. As a result, larger studies correlating developmental delay and/or autism spectrum disorder and haploinsufficiency of these genes are required.

ADHD is a widely discussed phenotype in individuals with 22q11.2 deletion syndrome [16,32–35]. Five out of our sixteen cases (cases 2, 5, 12–14) carried an ADHD diagnosis. Cases 2 and 5 all shared LCR A deletions with RefSeq and OMIM genes as previously described. Cases 12–14 harbored LCR D-E deletions, suggesting potential pathogenic haploinsufficiency with associated genes in this region. RefSeq genes in this region include *BCRP2*, *LOC102724728*, *AM230B*, *GGT2*, *POM121L8P*, *LOC107987389*, *FAM230H*, *RIMBP3B*, *HIC2*, *TMEM191C*, *PI4KAP2*, *RIMBP3C*, *UBE2L3*, *YDJC*, *CCDC116*, *SDF2L1*, *MIR301B*, *MIR130B*, *PPIL2*, *YPEL1*, *MAPK1*, *PPM1F*, *PPM1F-AS1*, *TOP3B*, *PRAMENP*, *VPREB1, BMS1P20*, *ZNF280B*, *ZNF280A*, *PRAME*, and *LL22NC03-63E9.3*. Whether the deleted genes contribute to ADHD needs to be further investigated. Motahari et al. [20] mapped genes deleted in 22q11.2 LCR A-D to various cellular pathways and functions including chromatin modification/DNA replication, signaling, cell-cell adhesion, mitochondrial/metabolism, and transcription factors [20]. Such studies should lead to understanding of disease pathogenesis and associated genes and cellular pathways.

Interestingly, cases 2, 6, 10, 11 were associated with preterm birth; moreover, case 10 presented with lethality shortly after birth. Preterm and perinatal 22q11.2 deletion syndrome lethality has been previously studied [36]. This group reported a cohort study of perinatal outcomes in a group of infants harboring 22q11.2 deletions with a resulting lethality rate of 3/42. However, whether subregional deletions in the 22q11.2 locus are associated with varying frequencies is unclear and requires further investigations. Ultimately, retrospective case series with detailed 22q11.2 deletion locations and subsequent prospective investigations may be needed to better estimate lethality outcomes of 22q11.2 locus subregions. These findings may guide clinicians and families regarding CMA testing and perinatal outcomes.

Case 4 was originally believed to be Goldenhar syndrome. Goldenhar syndrome is characterized by facial asymmetry and pronounced facial defects, like microtia or anotia, benign ocular growths, and spinal abnormalities [37,38]. Goldenhar syndrome has been associated with 22q11.2 deletion syndrome [39,40]. It is interesting that our patient has a deletion in 22q11.2 LCR A whereas the reported cases harbor proximal, central, and distal deletions [39,40]. Goldenhar syndrome shows significant locus heterogeneity and has been associated with copy number variations (CNVs) at multiple chromosome regions including Xp22.33p22.31, 1p22.2p31.3, 2p11.2, 2p12, 2q11, 3q29, 4p16.3p15.33, 5p15, 5q22, 8q13.3, 9p22.1, 10q26.2q26.3, 12p13.33, 13q34, 14q23.1, 14q31.1q31.3, 15q24, 16p13.3, 17q11.2, 22q11.1, 22q11.1q11.21, and 22q11.2 [39,40]. Further investigation into the genotype and phenotype association of Goldenhar syndrome should be considered [39,40].

The phenotypic contributions of genes and regions in the central and distal 22q11.2 deletions have had very limited outcome studies [16,41]. Future investigations involving these regions and their corresponding phenotypes would shed light to the degree of pathogenicity of regional 22q11.2 deletions. Possible studies include retrospective and prospective cohort studies of central and distal 22q11.2 deletions to determine both neonatal and developmental phenotypes. Case control studies would also help determine if these phenotypes are secondary to 22q11.2 deletions or if they are background findings related to polygenic or multifactorial mechanisms as discussed by other studies [8,42–44]. Further, translational studies in murine models may have utility as these have been demonstrated to be associated with the partner 22q11.2 duplication neurodevelopmental syndromes in central and distal segments [12]. Finally, continued reports of both healthy and impaired patients harboring 22q11.2 deletions is critical in determining penetrance of 22q11.2 phenotypes and prognosis of offspring in parents harboring 22q11.2 deletions.

## Figures and Tables

**Table 1.** Results of CMA and clinical features of the patients with 22q11.2 deletions.

Case no.	Gender	Age at diagnosis	Age at chart review	Deletion region	Deletion size (kb)	Flanking LCRs	Origin	Cardiovascular system	Skeletal system	Gastrointestinal system	Pulmonary system	Immune system	Other
1	M	8 y	12 y	chr22: 18,916,842–19,024,794	108	A	NA	–	Gait abnormalities	–	–	–	Obesity; Hearing loss
2	M	11 m	3 y	chr22: 18,916,842–19,024,659	108	A	NA	–	–	–	–	Frequent infection	Anemia of prematurity; Retinopathy; Wide spaced nipples; Spicanthal folds
3	F	3 y	8 y	chr22: 18,916,842–19,024,659	108	A	NA	–	–	–	–	Allergic rhinitis; Recurrent ear infection	–
4	F	2 m	5 y	Chr22: 18,916,842–19,024,659	108	A	NA	Secundum ASD with spontaneous closure	Polydactyly	–	–	Recurrent otitis media	Goldenhar syndrome
5	M	1 m	6 y	Chr22: 18,644,790–21,465,659	2,821	A-D	NA	Membranous VSD; Secundum ASD; Trace tricuspid insufficiency; Murmur	–	–	B/L peripheral pulmonary stenosis	SCID; Recurrent oral thrush; Low TREC at birth	Pierre Robin sequence; Poor weight gain; B/L middle ear disorder
6	M	5 y	9 y	Chr22: 18,916,842–21,465,662	2,548	A-D	NA	Murmur not present at birth	–	–	–	Positive ANA, Low T cell count; Recurrent ear infection	Hypo-developed scrotum; Polycystic kidney disease; Glandular hypospadias; Obesity
7	F	3 y	10 y	Chr22: 18,916,842–21,800,797	2,884	A-D	NA	Tetralogy of Fallot	–	–	–	Low lymphocyte count at birth	–
8	M	6 y	12 y	Chr22: 18,916,842–21,800,797	2,884	A-D	NA	Secundum ASD, trivial mid-muscular VSD, Thickening of Aortic valve with trace regurgitation	–	–	–	–	Hearing loss
9	M	14 d	2 y	Chr22: 18,916,842–21,915,509	2,999	A-D	NA	Supraventricular tachycardia; ASD with spontaneous closure	Polydactyly	Born with inguinal hernia; Gastrostomy tube after birth; Runny stools; Flatulence	Respiratory distress at birth; Tracheotomy	Low TREC at birth; Hypoparathyroidism; Low lymphocytes	Renal pyelectasis; U/L middle ear dysfunction; Paralysis of true vocal cords
10	F	Died at 8 d	8 d	Chr22: 18,916,842–21,800,797	2,884	A-D	NA	Cardiac failure; Intraventricular hemorrhage of left; Moderate size secundum ASD; Moderate size patent ductus arteriosus; Mild-moderate right atrial and right ventricular dilatation; Trace tricuspid insufficiency	–	–	Hypoplastic lungs with pulmonary hypotension; Respiratory failure	–	B/L multicystic dysplastic kidneys; Hypocalcemia; Hypomagnesemia; Hypopotassemia; Thrombocytopenia
11	F	8 d	1 y	Chr22: 18,648,866–21,800,797	3,152	A-D	Assumed maternal	Moderate-large ASD; Mild right atrial and ventricular enlargement with thickening of aortic valve	Microcephal; Short stature	Born with inguinal hernia; Gastrostomy tube for poor feeding; Recurrent GERD	Cyanotic respiratory distress at birth	Low IgM levels	Hypocalcemia
12	M	6 m	4 y	Chr22: 21,049,799–21,798,907	749	C-D	NA	Murmur; Trace tricuspid insufficiency; Dilated coronary sinus; Persistent left vena cava	–	Occasional constipation	–	Hashimoto thyroiditis; Elevated TSH; Recurrent oral candida; Low T cell at birth	B/L renal pyelectasis
13	F	2 y	9 y	Chr22: 21,465,661–22,962,196	1,497	D-E	NA	Secundum ASD; Trace tricuspid insufficiency	–	Chronic constipation	–	Frequent respiratory infection	–
14	M	1 y	7 y	Chr22: 21,465,661–22,962,196	1,497	D-E	Maternal	Murmur	Low muscle tone; Leg length discrepancy with outturned leg and abnormal gait	Some constipation with green stool	–	Low WBC count; Recurrent stuffy nose; Recurrent URIs	Single palmar crease; Hearing loss; B/L ear tags; Eczema
15	F	24 y	30 y	Chr22: 21,465,661–22,962,196	1,497	D-E	NA	–	–	–	–	Allergic rhinitis	Seborrheic dermatitis
16	F	2 d	6 m	Chr22: 22,962,196–23,649,155	687	E-F	NA	Complete atrioventricular septal defect; Large primum ASD; Large endocardial cushion; VSD; Single common thickened and dysplastic atrioventricular valve with moderate regurgitation; Mild thickening of the aortic and pulmonary valves; Pulmonary insufficiency	NA	NA	NA	Transient abnormal myelopoiesis associated with Down syndrome	NA

Notes: Genomic linear positions are given relative to NCBI build 37 (hg19); –, indicates no abnormal features reported in the patient; ASD, atrial septal defect; ANA, antinuclear antibody; B/L, bilateral; d, days; F, female; GERD, gastroesophageal reflux disease; kb, kilo base pairs; IgM, immunoglobulin M; M, male; m, months; NA, information is not available; SCID, Severe combined immunodeficiency; TREC, T cell receptor excision circle; TSH, thyroid-stimulating hormone; U/L, unilateral; URI, upper respiratory infection; VSD, ventricular septal defect; WBC, white blood cell; y, years old.

**Table 2.** Birth histories and neurodevelopmental and psychiatric disorders in patients with 22q11.2 deletions.

Case no.	Birth history	Speech delay	Growth delay	Motor delay	Intellectual disability	Psychiatric disorder
1	NA	At 4 y	–	Fine motor delay at 11 y	At 4 y	ADHD, ASD, anxiety, irritability at 4 y
2	Born at 31 w; Developed amenia of prematurity and retinopathy	–	–	Gross motor delay at 7 m	–	–
3	SVD at 39 w with abnormal umbilical cord insertion	At 3 y	–	Fine motor and gross motor delay at 3 y	–	ADHD, ASD, sleep disorder and anxiety at 2 y
4	–	–	–	–	–	–
5	–	Borderline communication delay at 4 y	Poor weight gain in infancy	Fine motor and gross motor delay at 4 y	–	–
6	Born at 30 w	At 5 y	Hypotonia and failure to thrive in childhood	Fine motor and gross motor delay at 5 y; Walked at 18 m	Unspecified intellectual delay	ADHD, anxiety, irritability at 5 y
7	Borderline small for gestational age	At 4 y	–	–	–	–
8	Stayed in hospital after birth for 1 y with respiratory infections	Noted delay at 6 m; Spoke in single words until 3 y	–	Hypotonia; Did not sit until 1 y; Walked at 2.5 y; Fine motor and gross motor delay at 7 y	At 7 y	–
9	Echogenic cardiac foci on ultrasound; Tracheomalacia; Macrosomia; Hypoparathyroidism; Hypocalcemia; Asthma; Anemia of newborn; Weak cry	Mixed receptive-expressive disorder at 8 m	–	Fine motor and gross motor delay at 2 m	–	–
10	Delivered at 35 4/7 w by C-section; Anemia, spontaneous pneumothorax; Respiratory distress; Died at 8 d	NA	NA	NA	NA	NA
11	Delivered at 32 1/2 w by C-section; Born cyanotic in respiratory distress; Resuscitated after 3 minutes	–	–	–	–	Seizure like activity in NICU
12	Mother used tobacco and cannibis during pregnancy; Delivered by C-section; Child needed resuscitation at birth	–	Poor weight gain at 15 m	–	–	–
13	Delivered by C-section	At 2 y	Small for age at 2 y; Slow weight gain	Fine motor and gross motor delay at 2 y	–	ADHD and ASD at 2 y
14	Born at 37 w; Slight jaundice after birth	Mixed receptive-expressive language disorder at 13 m	At 6 y	Fine motor and gross motor delay at 13 m	At 6 y	ADHD at 5 y
15	NA	–	–	–	–	ADHD
16	Anemia; Persistent pulmonary hypertension of newborn	NA	NA	NA	NA	NA

Notes: –, no abnormal features reported in the patient; ADHD, attention-deficit hyperactivity disorder; ASD, C-section, cesarean section; m, month; NA, information is not available; NICU, neonatal intensive care unit; SVD, spontaneous vaginal delivery; y, year.

**Table 3.** Craniofacial and velopharyngeal features of the patients with 22q11.2 deletions.

	Case 1	Case 2	Case 3	Case 4	Case 5	Case 6	Case 7	Case 8	Case 9	Case 10	Case 11	Case 12	Case 13	Case 14	Case 15	Case 16
Deletion regions	A	A	A	A	A-D	A-D	A-D	A-D	A-D	A-D	A-D	C-D	D-E	D-E	D-E	E-F
Hypertelorism							+	+						+		
Frontal Bossing			+		+											
Low set ears								+								
Micrognathia				+	+											
Cleft palate/VPI		S			S	S		V								
Small head circumference at birth <5th centile		+									+					
Small head circumference at 28 months <5th centile											+					
High palate arch				+			+									
Ear canal atresia														+		
Periauricular tag				+										+		
Brachycephalic														+		
Retrognathia			+													
Malocclusion			+													
Glottic web									+			+				
Upslanting palpebral fissure																+
Dysmorphic facies						+										
Prominent nasal bridge														+		
Microtia				+												
Almond shaped eyes																
Thin upper lip																
Smooth philtrum																

Notes: S, submucous cleft palate; VPI/V, velopharyngeal insufficiency.

**Table 4.** Common phenotypic features for cases with different deletions.

Phenotypic features	Proximal	Central	Distal
	This study (A-D)	Burnside 2015 (A-B, A-D)	This study (C-D)	Burnside 2015 (B-D, C-D)	This study (D-E)	Burnside 2015 (C-E, D-E, D-F)
Cardiovascular defects	7/7 (100%)	~315/426 (74%)	1/1 (100%)	20/101 (20%)	2/3 (66%)	24/45 (53%)
Skeletal anomalies	2/7 (29%)	>64/426 (15%)	–	12/68 (18%)	1/3 (33%)	22/45 (49%)
Gastrointestinal anomalies	2/7 (29%)	~153/426 (36%)	1/1 (100%)	3/68 (4%)	2/3 (66%)	10/45 (22%)
Immune deficiency/recurrent infections	5/7 (71%)	~328/426 (77%)	1/1 (100%)	10/68 (15%)	3/3 (100%)	9/45 (20%)
Language delay	5/6 (83%)	Common	–	15/68 (22%)	2/3 (66%)	6/45 (13%)
Developmental delay	4/6 (67%)	Common	1/1 (100%)	16/68 (24%)	2/3 (66%)	21/45 (47%)
Intellectual disability	2/6 (33%)	~298/426-383/426 (70-90%)	–	17/68 (25%)	1/3 (33%)	18/45 (40%)
Psychiatric disorder	2/6 (33%)	~256/426 (60%)	–	12/68 (18%)	3/3 (100%)	13/45 (29%)
Craniofacial anomalies	6/7 (86%)	Common	1/1 (100%)	31/68 (46%)	1/3 (33%)	26/45 (58%)

Notes: source: Burnside RD (2015) [1].
